# Supplementary material for: WordCommentsAnalyzer: A windows software tool for qualitative research
Source: F1000Res. 2018 Sep 4;7:536. Originally published 2018 May 3. [Version 2] doi: 10.12688/f1000research.14819.2 (PMC6137411; doi:10.12688/f1000research.14819.2)
Supplement: Supplementary file 3 [file f1000research-7-17614-s0002.tgz › b2a79496-4ada-47c4-9682-a99ce867836d.zip!/Eight Iranian Health Journals/Journal of Health System Research _25.docx]

© Journal of Health System Research . This work is licensed under CC BY-NC 4.0 (https://creativecommons.org/licenses/by-nc/4.0/).

Volume & Issue: دوره 9، شماره 7؛1392

Title: بررسی ارتباط اختلال عملکرد جنسی و سطح افسردگی در مردان مبتلا به هپاتیت مزمن

http://hsr.mui.ac.ir/index.php/jhsr/article/view/857

مقدمه: اختلال عملکرد جنسی به عنوان یکی از مشکلات بهداشتی مهم تلقی می‌شود. بیماری‌های مزمن از جمله هپاتیت مزمن و درمان آن می‌تواند تأثیر منفی بر روی خلق و عملکرد جنسی داشته باشد. این پژوهش با هدف تعيين ارتباط اختلال عملکرد جنسی با سطح افسردگی در بیماران مرد مبتلا به هپاتیت مزمن صورت گرفته است. روش‌ها: این مطالعه توصیفی تحلیلی از نوع همبستگی بر روی 70 بیمار مبتلا به هپاتیت مزمن که ویژگی‌های ورود به مطالعه از قبیل: متاهل بودن، عدم ابتلا به اختلال عملکرد جنسی قبل از تشخیص هپاتیت، عدم استفاده از داروهای مؤثر بر اختلال عملکرد جنسی، عدم ابتلا به بیماری‌های تأثیرگذار بر اختلال عملکرد جنسی را داشتند، انجام شد. روش نمونه‌گیری ساده و در دسترس بود. برای بررسی وضعیت عملکرد جنسی بیماران از پرسشنامۀ بین‌المللی استاندارد اختلال عملکرد جنسی(international index of erectile function) استفاده شد. جهت تعیین روایی از روایی محتوا و برای پایایی آن از آزمون مجدد استفاده شد. جهت ارزیابی سطح افسردگی، از پرسشنامه استاندارد بک(Beck) استفاده گردید. داده‌ها، توسط نرم‌افزارSPSS16  تجزیه و تحلیل شد. یافته‌ها: متوسط سن بیماران 56/45 سال و میانگین مدت زمان ابتلا به بیماری 81/6 سال بود. 7/65% مبتلا به هپاتیت مزمن B و3/34%  مبتلا به هپاتیت مزمنC بودند. بیشترین درصد بیماران (3/74%) اختلال نعوظ متوسط و 6/8% دارای اختلال نعوظ ضعیف بودند. شدت افسردگی در40% از بیماران خفیف بود. بین سطوح مختلف افسردگی از نظر اختلال نعوظ اختلاف معنی‌داری وجود نداشت (449/0=P). بین اختلال نعوظ و مشخصات دموگرافیک نیز ارتیاطی یافت نشد. نتیجه‌گیری:  بین اختلال نعوظ وسطوح افسردگی در بیماران مبتلا به هپاتیت مزمن ارتباطی وجود ندارد.

Title: بررسی عوامل موثر بر خودسوزی در بین زنان (مطالعه موردی شهرستان‌های روانسر و جوانرود استان کرمانشاه)

http://hsr.mui.ac.ir/index.php/jhsr/article/view/872

مقدمه: خودکشی از جمله آسیب‌های اجتماعی است که در بیشتر جوامع دیده می‌شود. شیوه‌های خودکشی نیز موضوعی مهم است که در این بین خودسوزی از دردناک‌ترین روش‌ها است. خودسوزي تحت تاثیر عوامل مختلفی انجام می‌گیرد. این پژوهش با هدف مطالعه پدیده خودسوزی در بین زنان شهرستان‌های روانسر و جوانرود کرمانشاه و ویژگی‌های جمعیت‌شناختی قربانیان ناموفق خودسوزی انجام شد. روش‌ها: این پژوهش از نوع پیمایشی - توصيفي بود و به روش كيفي و برساخت‌گرایی انجام شد. مشاركت‌كنندگان در تحقيق 16 نفر از زنان نجات يافته از خودسوزی در مناطق شهری و روستایی منطقه مورد تحقیق بودند.  نمونه‌گيري به روش مبتنی بر هدف انجام شد و تا اشباع داده‌ها ادامه يافت. داده‌ها نیز از طریق  مصاحبه عميق و نیمه ساخت‌یافته جمع‌آوری شد. یافته‌ها: نتایج کلی حاکی از آن است که اکثر پاسخگویان متاهل(3/56%) ، روستایی(5/62) ، خانه‌دار(3/56%) و دارای تحصیلات ابتدای(50%) بوده‌اند.خودسوزی بیشتر در گروه سنی 30-16 سال مشاهده شد. دلیل عمده خودسوزی از دید پاسخگویان (44 %) نشان دادن شهامت و وخامت اوضاع فرد عنوان شده است. نتیجه‌گیری: براساس نتایج معتقدیم دلیل عمده خودسوزی در جامعه مورد مطالعه تفاوت ارزشی و هنجاری بین نسل‌هاست. لذا ضمن فرآیندی، اجتماعی و روانی دیدن این پدیده، ضروری است اقدامات مناسبی از جمله انجام تحقیقات بیشتر و همکاری سازمانهای ذیربط در این خصوص، ارائه آموزش های مناسب جهت  کاستن از باورهای سنتی و متعصبانه ، فراهم آوردن بسترهای مناسب تحصیلی و شغلی برای زنان منطقه، پر کردن اوقات فراغت این قشر به نحوی مناسب جهت جلوگیری از یکنواختی زندگی،افسردگی و نامیدی.جهت کاهش این آسیب اجتماعی صورت گیرد

Title: بررسی ارتباط بین استرس شغلی با رضایت شغلی در بین ماماهای شاغل در زایشگاه‌های شهر اصفهان

http://hsr.mui.ac.ir/index.php/jhsr/article/view/873

مقدمه: استرس شغلی یکی از عوامل مهم ایجاد عوارض جسمی و روانی و همچنین نارضایتی شغلی در ماماها و در نتیجه کاهش کیفیت خدمات ارایه شده می‌باشد. از آنجا که سلامت جسمی و روحی ماما به طور مستقیم و غیرمستقیم می‌تواند روی سلامت مادران و نوزادان تأثیر بگذارد، این مطالعه به منظور بررسی رابطه بین استرس شغلی و رضایت‌مندی شغلی ماماهای شاغل در بخش زایشگاه صورت گرفت. روش‌ها: این مطالعه توصیفی تحلیلی از نوع مقطعی می‌باشد .جامعه پژوهش شامل 106 مامای شاغل در زایشگاه‌های شهر اصفهان است. روش جمع‌آوری اطلاعات، پرسشنامه‌ بود و داده‌ها با استفاده از نرم‌افزار SPSS نسخه 20 و آزمون‌های آماری توصیفی و استنباطی (آنالیز واریانس یک‌طرفه، همبستگی پیرسون و اسپیرمن و آزمون t مستقل) مورد تجزیه و تحلیل قرار گرفتند. یافته‌ها: نتایج این مطالعه نشان داد که میانگین نمره استرس شغلی ماماها 8/25 ± 41 از نمره 100 و میانگین نمره رضایت شغلی آنها 43/16± 75/44 می‌باشد. آزمون آماری ضریب همبستگی پیرسون ارتباط معناداری بین استرس شغلی و رضایت‌مندی شغلی ماماها نشان داد (001/0>P و 806/0-=r ). همچنین بین میزان استرس شغلی و جنبه‌های مختلف رضایت شغلی نیز رابطه معنادار وجود دارد (05/>P). در این مطالعه بین استرس شغلی و رضایت کلی شغلی با مشخصات فردی مثل سن و وضعیت تأهل و میزان سابقه کاری و میزان تحصیلات رابطه معنادار وجود نداشت، در صورتی که بین استرس شغلی و رضایت کلی شغلی با میزان حقوق و مزایا و وضعیت استخدامی رابطه معنادار دیده شد (05/0>P). نتیجه‌گیری: با توجه به یافته‌های این پژوهش پیشنهاد می‌شود که مدیران برنامه‌ریزی سازمان‌های بهداشتی درمانی با به کارگیری استراتژی‌هایی به منظور کاهش میزان استرس و پیشگیری از آثار روانی و جسمانی آن بر شاغلین حرفه مامایی و در نتیجه افزایش میزان رضایتمندی شغلی آنها اقدام کنند که این می‌تواند گامی در جهت افزایش بهره‌وری سازمان‌های بهداشتی‌درمانی و همچنین کیفیت خدمات ارایه شده به دو قشر آسیب‌پذیر جامعه یعنی مادران و نوزادان باشد.

Title: تعیین و مقایسه اضطراب امتحان و منبع کنترل در دانشجویان دانشگاه پیام نور شهرستان های داران و چادگان

http://hsr.mui.ac.ir/index.php/jhsr/article/view/892

مقدمه: اضطراب امتحان عامل ضعف سیستم ایمنی بدن، بازدارنده پيشرفت تحصيلي، کاهش کارآمدی تحصیلی، و تحميل هزینه‌های زیاد بر جوامع است. منبع کنترل، چگونگی جست و جو و شیوه پردازش اطلاعات در محیط در رابطه با وقایع ومسایل زندگی است. پژوهش حاضر با هدف تعیین و مقایسه اضطراب امتحان و منبع کنترل درونی و بیرونی در دانشجویان دانشگاه پیام نور شهرستان‌های داران و چادگان استان اصفهان) در سال 1390 صورت پذیرفت. روش‌ها: این مطالعه توصیفی از نوع مقطعی بود که تعداد 288 نفر دانشجو به روش تصادفی با انتساب متناسب انتخاب و وارد مطالعه گردیدند. ابزار گردآوری اطلاعات دو پرسشنامه استاندارد اضطراب اشپیلبرگر و منبع کنترل راتر به همراه بخش اطلاعات جمعیت‌شناختی بود که به صورت خود گزارش‌دهی تکمیل گردیدند. داده‌ها توسط نرم‌افزار آماری SPSS نسخه 18 و  به وسیله آمار توصیفی و آزمون‌های تحلیلی نظیر T-test، ANOVA، Mann-Whitney U، Kruskal-Wallis H و Chi-Square در سطح معناداری 05/0 مورد تجزیه و تحلیل قرار گرفتند. یافته‌ها: میانگین سن دانشجویان 06/4±26/22 سال، 1/67% زن، 3/28% متاهل، 2/70% ساکن شهر بودند. 7/21% دارای منبع کنترل درونی، 3/78% بیرونی و 4/3% دارای اضطراب خفیف، 6/30% متوسط به پایین، 2/51% متوسط به بالا، 8/12% نسبتا شدید، 2/1% دارای اضطراب شدید بودند. میان سطح اضطراب دانشجویان و منبع کنترل ارتباط آماری معناداری وجود نداشت. دانشجویان ساکن شهر اکثرا دارای منبع کنترل بیرونی، مجردها دارای اضطراب بیشتری نسبت به متأهلین و با بالاتر رفتن سن میزان اضطراب کاسته می‌شد. نتیجه‌گیری: در اين مطالعه در بیش از نیمی از دانشجويان اضطراب با سطح متوسط به بالا وجود داشت، با توجه به اثرات منفي اين اضطراب بر عملکرد تحصيلي دانشجويان، اجراي برنامه‌های پيشگيري از اضطراب، ارایه خدمات روان‌شناختي و مشاوره و همچنين با توجه به بی‌ارتباط بودن منبع کنترل دانشجویان با سطح اضطراب آنها، شناسايي دیگر علل مرتبط با آن و سپس برنامه‌ريزي جهت مرتفع نمودن آنها، پيشنهاد می‌شود.

Title: بررسی ارتباط سلامت ‌معنوی با اضطراب و برخی مشخصات دموگرافیک بیماران عروق کرونر

http://hsr.mui.ac.ir/index.php/jhsr/article/view/893

مقدمه: بیماران عروق‌کرونر در مرحله حاد، اضطراب زیادی را تجربه ‌می‌کنند و نسبت به بیماران با اضطراب کمتر به میزان بالاتری به ایسکمی، سکته مجدد، تندتپشی و فیبریلاسیون ‌بطنی مبتلا می‌شوند. از طرفی بیماری‌های قلبی بر معنویت فرد تاثیر می‌گذارد. هر چند برخی مطالعات نشان‌داده‌اند، بیمارانی که نمره بالاتری در مقیاس‌های معنوی دارند میزان عوارض‌ قلبی‌ عروقی کم‌تری داشته‌اند؛ اگر چه در این میان یافته‌های متناقض به چشم می‌خورد؛ به این ترتیب محققین بر‌ آن‌ شد‌ند مطالعه‌ای را با هدف بررسی ارتباط‌ بین سلامت‌ معنوی‌ با اضطراب بیماران‌ عروق‌ کرونر طراحی و اجرا نمایند‌. روش‌ها: این مطالعه از نوع توصیفی- همبستگی بود. در طی 6 ماه مطالعه، 292 بیمار مبتلا به بیماری عروق کرونر، بستری در بخش ویژه به روش سرشماری، به عنوان جامعه آماری انتخاب شدند. از این تعداد، 85 نفر از بیماران که معیارهای ورود به مطالعه را داشتند، پرسشنامه‌ها (اطلاعات‌ دموگرافیک، سلامت‌ معنوی و اضطراب‌ موقعیتی اسپیل‌برگر) را تکمیل نمودند. داده‌ها در مجموع از نوع کمی و کیفی بود که برای تجزیه و تحلیل آن‌ها از نرم‌افزار  SPSSنسخه 18 و روش‌هاي آماري توصيفي و ضریب همبستگی پیرسون و اسپیرمن استفاده گردید. در این مطالعه مقدار P در سطح کمتر از 05/0 معنا‌دار در نظر گرفته شد. یافته‌ها: به ترتیب میانگین نمره‌ کلی ‌سلامت‌ معنوی 68/9±11/103 از 120، میانگین نمره‌ وجودی‌ سلامت ‌معنوی 06/9±48/45 از 60 و میانگین نمره ‌مذهبی ‌سلامت ‌معنوی20/4±63/57 از 60 بود. همچنین یافته‌ها نشان داد میانگین نمره اضطراب موقعیتی بیماران 03/13±‌80/40 بود. یافته‌های مطالعه حاکی از معنادار شدن ارتباط بین سلامت معنوی و ابعاد آن با میزان اضطراب بیماران ایسکمیک قلبی بود، به‌ طوری‌که آزمون پیرسون نشان‌داد ارتباط آماری معنی‌دار معکوس بین نمره‌کلی‌ سلامت ‌معنوی با اضطراب (001/0P< و 47/0=- r)، نمره ‌بعد ‌وجودی سلامت‌ معنوی با اضطراب (001/0P< و 46/0= - r) و نمره بعد مذهبی‌ سلامت ‌معنوی با اضطراب (01/0=P و26/0=  r ) وجود داشت. نتیجه‌گیری: این مطالعه قویا نشان داد در بیماران عروق کرونر با نمرات بالاتر سلامت‌ معنوی، میزان اضطراب کمتری وجود دارد و بالا بودن سلامت ‌معنوی یک فاکتور مهم محافظت‌کننده در برابر بروز اضطراب در این بیماران است.

Title: بررسی آگاهی و نگرش مادران در زمینه بهداشت دهان و دندان کودکان پیش دبستانی درشهراصفهان

http://hsr.mui.ac.ir/index.php/jhsr/article/view/956

مقدمه: پوسیدگی دندان شايع‌ترين بيماري مزمن دوران کودکي مي‌باشد و خانواده مهمترين نقش را در رعايت بهداشت دندان و دهان کودکان دارند. این مطالعه با هدف بررسی آگاهی و نگرش مادران در زمینه بهداشت دهان و دندان کودکان پیش دبستانی انجام شد.  روش‌ها : مطالعه از نوع مقطعی بود و به روش نمونه‌گیری تصادفی بر روی 253 نفر از مادران دارای کودک پیش دبستانی (6-3 ساله) مراجعه‌کننده به مراکز بهداشتی درمانی شهر اصفهان انجام گرفت. اطلاعات به وسیله پرسشنامه‌ای که روایی محتوایی و پایایی آن مورد تایید قرار گرفته بود، جمع آوری شد و برای تحلیل یافته‌های این پژوهش از آزمون‌های آماری t مستقل ،ضریب همبستگی اسپیرمن و پیرسون استفاده و 05/0 p <نیز به عنوان سطح معنی‌دار در نظر گرفته شده. یافته‌ها: میانگین سن مادران 5 ± 9/28 سال (دامنه20-48) محاسبه گردید. تعداد 37 نفر از (6/14%) مادران شاغل و 216 (4/85%) نفر خانه‌دار بودند. میزان آگاهی 2/14% مادران در خصوص بهداشت دهان و دندان کودکان مطلوب و 7/61% متوسط بود. 6/10% از مادران نگرش متوسط و 4/89% نگرش مطلوب داشتند. آزمون همبستگی اسپیرمن نشان داد که بین نمره آگاهی با سطح تحصیلات رابطه مستقیم وجود دارد (001/0P < و 516/0 = r) همچنین ارتباط معنی‌داری بین آگاهی و نگرش مادران مشاهده شد (001/0P <). نتیجه‌گیری: در این مطالعه اکثر مادران آگاهی متوسط و ضعیفی در زمینه بهداشت دهان و دندان کودکان پیش دبستانی داشتند. بنابراین برنامه‌ریزی آموزشی جهت بالا بردن سطح آگاهی مادران ضروری به نظر می‌رسد.

Title: ارزيابي ارگونوميك محیط کار و بررسی شیوع آسیب‌های اسكلتي- عضلاني در یک شرکت ساخت و تولید ژنراتور

http://hsr.mui.ac.ir/index.php/jhsr/article/view/967

مقدمه: اختلالات اسکلتي- عضلاني، يکي از عوامل شايع آسيب‌هاي شغلي است که به طور عمده در کمر، گردن و اندام‌هاي فوقاني نمايان مي‌شود. این مطالعه با هدف تعیین شیوع اختلالات اسکلتی- عضلانی و ارزیابی ارگونوميك خطر ابتلا به این اختلالات در کارکنان یک شرکت مهندسی و ساخت ژنراتور انجام شده است. روش‌ها: در این مطالعه 319 نفر از کارکنان یک شرکت مهندسی و ساخت ژنراتور با حداقل یک سال سابقه کار مورد بررسی قرار گرفتند. در مشاغل ستادی ابزار گردآوری داده‌ها عبارت بودند از پرسشنامه ویژگی‌های دموگرافیک، پرسشنامه نوردیک و چک لیست ارزیابی ارگونومیک محیط‌های کار ستادی. در مشاغل تولیدی ابزار گردآوری داده‌ها شامل پرسشنامه ویژگی‌های دموگرافیک، پرسشنامه نوردیک و روش Quick Exposure Check   (QEC) برای ارزیابی ریسک ابتلا به اختلالات اسکلتی- عضلانی بودند. يافته‌ها: میانگین سن و سابقه کار افراد مورد مطالعه به ترتیب برابر با 05/7±84/35 و 98/4±54/7 سال به دست آمد. شیوع اختلالات اسکلتی- عضلانی در کارکنان ستادی و تولیدی به ترتیب 5/79 و 9/82% تعیین شد. در ارزیابی به وسیله‌ی چک لیست ارگونومی در کارکنان ستادی مشخص گردید بهبود ایستگاه کار می‌بایست در اولویت قرار گیرد. در ارزیابی به روش QEC، در مجموع 6/83% از موارد سطح ريسك بالا و بسيار بالا بود كه نشان‌دهنده مخاطره‌آميز بودن شرایط و نیاز به اصلاحات در مشاغل تولیدی مي‌باشد. نتیجه‌گیری: در بهبود شرایط کار و اقدامات اصلاحی در افراد مورد مطالعه، توجه به ریسک فاکتورهای نواحی کمر، گردن، زانو، پشت و شانه به علت شیوع بالا در این نواحی اهمیت دارد.

Title: بررسی ارتباط مولفه‌های جو سازماني با ابعاد تعهد سازماني در پرستاران شاغل بیمارستان‌های منتخب دانشگاه علوم پزشکی تهران

http://hsr.mui.ac.ir/index.php/jhsr/article/view/982

مقدمه: یکی از عوامل موثر بر تعهد سازمانی جو سازمانی است. جو سازمانی ضمن تاثیر بر سلوک اعضاء، رفتار سازمانی آنها را نیز تحت تاثیر قرار می‌دهد.این مطالعه با هدف تعیین ارتباط مولفه‌های جو سازمانی و ابعاد تعهد سازمانی پرستاران شاغل در بیمارستان‌های منتخب دانشگاه علوم پزشکی تهران در سال 1390 طراحی و اجرا شد. روش‌ها: پژوهش حاضر یک مطالعه توصيفي ـ تحليلي از نوع مقطعی و کاربردی بود. واحدهای پژوهش این مطالعه 363 پرستار کارشناس و کارشناس ارشد شاغل در بیمارستان‌های منتخب دانشگاه علوم پزشکی تهران بودند که به شیوه تصادفی انتخاب شدند. ابزار جمع‌آوری اطلاعات پرسشنامه روا و پایا در سه بخش اطلاعات فردی، تعهد سازمانی و جو سازمانی بود که به صورت خود ایفا تکمیل نمودند. برای تجزیه و تحلیل داده‌ها از نرم افزارآماریSPSS  نسخه 5/11 وآزمون‌های آمار توصیفی ( میانگین و انحراف معیار برای متغیر تعهد سازمانی) و به دلیل توزیع غیر نرمال ابعاد جو سازمانی از شاخص‌های پراکندگی مانند میانه و دامنه میان چارکی و ضریب همبستگی اسپیرمن به کار گرفته شد. سطح معنی‌داری در این مطالعه کمتر از 05/0 در نظر گرفته شد. .يافته‌ها: تعهد سازمانی پرستاران 15/32±87/65 و کلیه ابعاد آن (عاطفی، مستمر و تکلیفی ) در سطح متوسط قرار داشت. همچنین، از هشت مولفه متغیر جو سازمانی میانه و دامنه میان چارکی مولفه روحیه گروهی در سطح بالا و میانه سایر مولفه‌های سطح سازمانی در سطح متوسط قرار داشت. ارتباط‌سنجی این دو متغیر نشان داد که تعهد سازمانی با مولفه‌های روحیه گروهی ( 001/0>P  و29/0=r )، علاقمندی (001/0>P  و294/0=r)، صمیمیت (018 /0=P  و124/0=r)، ملاحظه‌گری (001/0>P  و362/0=r )، فاصله‌گیری (001/0=P  و175/0=r)، نفوذ و پویایی (001/0>P  و431/0=r ) و تاکید بر تولید ( 001/0>P  و384/0=r )رابطه خطی و مستقیم ومعنی‌دار و با مولفه مزاحمت (001 /0>P  و257/0 - =r ) رابطه خطی غیر مستقیم و معنی‌دار دارد.  نتیجه گیری: با توجه به ارتباط کلیه مولفه‌های جو سازمانی با تعهد سازمانی، بنابراین تلاش برای بهبود جو سازمانی در بیمارستان‌ها می‌تواند به عنوان یک استراتژی ارزشمند برای بهبود تعهد سازمانی محسوب شود.

Title: بررسی وضعيت سقط و برخی عوامل موثر بر آن در مراجعین بیمارستان‌های شهر زنجان

http://hsr.mui.ac.ir/index.php/jhsr/article/view/1074

مقدمه: نسبت مرگ و میر مادران از عوارض بارداری و زایمان يكي از مهمترین شاخص‌هایی است که نشان‌دهنده توسعه کشورهاست. عوارض ناخوشایند و در بعضی موارد خطرناک سقط برای مادر غیرقابل انکار است. آگاهي از فراوانی سقط در اجتماع مي‌تواند معياري غيرمستقيم از وضعيت سلامت مادران باشد. اين مطالعه با هدف ارزيابي وضعيت سقط و برخی عوامل موثر بر آن در مراجعین بیمارستان‌های شهر زنجان در سال 1390 طراحي و اجرا شد. روش‌ها: اين مطالعه از نوع توصیفی- تحلیلی بوده که بر روي 545 نفر مراجعین باردار بیمارستان‌های آیت الله موسوی و امام حسین (ع) شهر زنجان در سال 1390 انجام شد. ابزار گردآوری اطلاعات پرسشنامه بود كه در چهار بخش. اطلاعات دموگرافیك(11 سؤال)، اطلاعات مربوط به سابقه مامایی(12 سؤال) ، علل احتمالی سقط و اطلاعات مربوط به سقط اخیر از قبیل نوع، روش و محل انجام سقط (16 سؤال) تنظیم شد. داده‌های جمع‌آوری شده با استفاده از نرم افزار SPSS-11.5 و به كارگيري آزمون‌هاي توصيفي (ميانگين و انحراف معيار) و استنباطي ( كاي دو و رگرسيون لجستيك)تجزیه و تحلیل گردید. يافته‌ها: در این مطالعه فراوانی سقط 1/12 %(66 مورد) بود که 5/9%(52 مورد) آن خود بخودی و 6/2 %(14 مورد) از نوع درمانی بود. بین سقط جنین با بیماری تیروئید (01/0 P=)، خواسته یا ناخواسته بودن بارداری (01/0P=)، تعداد بارداری‌ها (02/0 P=)، سن مادر (003/0 P=)، شغل مادر (001/0 < P)، سابقه سقط قبلی (001/0 < P)، فاصله دو بارداری اخیر (01/0 P=) و وقوع بارداری در حین پیشگیری (03/0 P=) اختلاف معنی‌دار وجود داشت. نتایج نشان داد متغیرهای شاغل بودن (03/0 P=)، داشتن یک بارداری (001/0 < P) و بارداری شکم اول (006/0 P=) نسبت برتری بالاتری در وجود سقط را نشان دادند. نتيجه‌گيري: با توجه به يافته‌هاي مطالعه، تبیین اهداف در راستاي بهداشت باروري و سلامت مادر و برنامه‌ریزی در جهت ارتقاء سلامت باروري ضروری به نظر می‌رسد. همچنین لازم است در برنامه سلامت مادران، به خانم‌های باردار با سابقه مثبت سقط خدمات مطلوب‌تري ارائه گردد.

Title: بررسی مقایسه‌ای اولویت‌های ارزشیابی اساتید از دیدگاه اعضای هیئت علمی و دانشجویان دانشگاه علوم پزشکی لرستان

http://hsr.mui.ac.ir/index.php/jhsr/article/view/1163

مقدمه: آموزش از عمده‌ترين رسالت‌هاي يك دانشگاه و از وظايف مهم اعضاي هيأت علمي بوده و ارتقاء كيفيت آن، منجر به ارتقاء كيفيت آموزش دانشگاه مي‌گردد. از فرآيندهايي كه به منظور اين ارتقاء كيفيت انجام مي‌گردد ارزشيابي مدرس است. این مطالعه با هدف مقایسه اولویت‌های ارزشیابی مدرسین از دیدگاه اساتید و دانشجویان دانشگاه علوم پزشکی لرستان در سال 1390 انجام گردید. روش‌ها: این مطالعه به صورت توصیفی تحلیلی مقطعی انجام گردید. ابزار مورد استفاده پرسشنامه محقق ساخته بود که از طریق آن، اهمیت هر سؤوال از 5 حیطه ارزشیابی اساتید به صورت لیکرت 4 گزینه‌ای در بین 299 نفر از دانشجویان و تعداد 116 نفر از اعضا هیأت علمی دانشگاه علوم پزشکی لرستان به روش نمونه‌گیری چند مرحله‌ای مورد ارزیابی قرار گرفت. اطلاعات جمع‌آوري شده، با استفاده از نرم‌افزار SPSS نسخه 18 و آمار توصیفی و آزمون مجذور كاي، تی مستقل و آنالیز واریانس یک‌طرفه با سطح معنی‌داری 05/0 مورد تجزیه و تحلیل قرار گرفت. یافته‌ها: در حیطه مهارت‌های تدریس، تسلط استاد بر موضوع درس، در حیطه ویژگی‌های فردی، داشتن اعتماد به نفس، در حیطه مهارت‌های ارتباطی، برقراری ارتباط صمیمانه با دانشجو، در حیطه رعایت اصول و قوانین آموزشی، رعایت زمان دقیق شروع و پایان کلاس و در حیطه مهارت‌های ارزشیابی، برگزاری امتحان جامع و دقیق در پایان ترم توسط دانشجویان به عنوان مهمترین موارد انتخاب شد. نظرات دانشجویان و اساتید در مورد اهمیت موارد ارزشیابی با هم تفاوت داشت. نظرات افراد در اکثریت حیطه‌ها در دانشجویان در دو جنس از نظر آماری اختلاف معنی‌دار نشان داد ( 001/ P =). نتیجه‌گیری: موارد ارزشیابی از دیدگاه اساتید و دانشجویان تفاوت‌هایی گاهاً اساسی دارند، گرچه دانشجویان عوامل مهم در ارزشیابی اساتید را به خوبی تشخیص می‌دهند اما در اولویت‌دهی به موارد از فاکتورهایی نظیر جنسیت، معدل تحصیلی، مقطع و رشته تحصیلی تاثیر می‌پذیرند که می‌تواند اعتبار ارزشیابی‌ها توسط دانشجو را مورد تردید قرار دهد.

Title: بررسي ميزان حاكميت اخلاق كار اسلامي در نظام سلامت دردانشگاه علوم پزشکی اصفهان

http://hsr.mui.ac.ir/index.php/jhsr/article/view/1085

مقدمه: بسیاری از رفتارها و اقدامات مدیران و کارکنان، متأثر از ارزش‌های اخلاقی بوده و ریشه در اخلاق دارد. در شرایط کنونی عدم رعایت برخی معیارهای اخلاقی، نگرانی‌های زیادی را در بخش‌های دولتی و غیردولتی به وجود آورده است. یکی از عمده‌ترین دغدغه‌های مدیران کارآمد در سطوح مختلف، چگونگی ایجاد بسترهای مناسب برای رعایت اصول اخلاقی و تقویت حس مسؤولیت و تعهد عوامل انسانی است. نظام سلامت جامعه که توسط دانشگاه‌های علوم پزشکی کشور اداره می‌شود نیز از این قاعده مستثنی نبوده و توجه به معیارهای اخلاقی در کار با رویکرد اسلامی از حساسیت ویژه‌ای برخوردار است. بنابراین در این مطالعه شاخص‌های اسلامی اخلاق کار در بین کارکنان نظام سلامت بررسی و کالبد شکافی شده است. روش‌ها: این مطالعه از نوع توصیفی-کاربردی بود. جمعیت مورد مطالعه 8280 نفر در 4 ساختار نظام سلامت بوده که بر اساس توزیع اولیه، تعداد نمونه 239 نفر برآورد و پرسشنامه در بین آنها توزیع شد که تعداد 198 مورد از آنها برگشت داده شد ( نرخ بازگشت 83%). برای جمع‌آوری ادبیات از روش کتابخانه‌ای و جهت تأیید یا رد فرضیات از پرسشنامه محقق ساخته متشکل از 20 سؤال بسته که بر اساس طیف لیکرت تنظیم شده بود، استفاده گردید. پایایی با استفاده از روش آلفای کرنباخ برابر با91% محاسبه شد. همچنین روایی صوری و محتوایی طی چندین مرحله در روش دلفی توسط صاحب‌نظران و متخصصان، مورد تایید قرار گرفت. در نهایت تجزیه و تحلیل داده‌ها با روش‌های توصیفی شامل فراوانی، میانگین و روش استنباطی به کمک آزمون‌های دو جمله‌ای، کای‌اسکور با استفاده از نرم‌افزار SPSS نسخه 16 انجام شد. یافته‌ها: در این مطالعه از نظر آماری میزان حاکمیت مولفه‌های اخلاق کار اسلامی در بین کارکنان دانشگاه علوم پزشکی اصفهان با استفاده از آزمون دوجمله‌ای مطلوب بود. هیچ یک از عوامل بر مولفه‌های شاخص‌های ایمان و پشتکار و جدیت تاثیرگذار نبوده، شاخص روابط سالم انسانی در محل کار بر عوامل جنسیت و رسته شغلی اثرگذار بوده و در نهایت شاخص رفتار مسؤولانه دینی تحت تاثیر عوامل جنس، سن، رسته شغلی و سابقه مدیریتی بوده است. نتیجه‌گیری: با توجه به اهمیت بررسی رفتارهای اخلاقی در سازمان‌ها به ویژه اخلاق کار اسلامی، بررسی تاثیر اخلاق کار در ایران با تأکید بر اصول اخلاق اسلامی و فرهنگ حاکم بر سازمان‌ها بسیار حایز اهمیت است. با توجه به نتایج این مطالعه، حاکمیت اخلاق کار اسلامی در بین کارکنان مطلوب بوده و عوامل جنسیت، سن، رسته شغلی، سابقه مدیریتی بر میزان مولفه‌های اخلاق کار اسلامی تأثیرگذار است.

Title: نگرش زنان شاغل به آوردن فرزند دوم وعوامل موثربرآن

http://hsr.mui.ac.ir/index.php/jhsr/article/view/1495

مقدمه: از آن جایی که باروری پدیده اجتماعی است، مشکلات و عوامل متفاوت ومتنوعی وجود دارد که می‌توانند آن را تحت تأثیر قرار داده و آن را کنترل نمایند. اشتغال زنان از جمله عوامل تأثیرگذار بوده و تمایل زنان به باروری را از لحاظ اجتماعی واقتصادی تحت الشعاع خود قرار می‌دهد. روش‌ها: این پژوهش به روش پیمایشی در شهر اصفهان و شهرستان‌های کاشان، نجف‌آباد، سمیرم و گلپایگان صورت گرفت، اطلاعات مورد نیاز از تعداد 610 نمونه شامل زنان ازدواج کرده و شاغل دارای یک فرزند 15-49 ساله از طریق پرسشنامه محقق ساخته جمع‌آوری شد. پایایی پرسشنامه با استفاده از آزمون آلفا کرونباخ مورد بررسی قرار گرفت .سپس با نرم‌افزارSPSS  نسخه 19 تجزیه و تحلیل شد. یافته‌ها: بیشتر جمعیت مورد بررسی در پایگاه اجتماعی و اقتصادی متوسط قرار داشتند. بیشتر پاسخگوها در سنین 20-24 ساله و همسران آنها در سنین 25-29 سال قرار داشتند. تحصیلات افراد شرکت کننده در این مطالعه بیشتر در حد لیسانس بود بیشتر آنها دارای فرزندانی کوچکتر از 4 سال بودند و تعداد فرزند مطلوبشان دو فرزند بوده است. اکثر آنها تمایل به داشتن فرزند دوم بودندو اعتقاد داشتند  بهتر است زوجین دارای دو فرزند باشند. تحصیلات، سن زنان، پایگاه اجتماعی و اقتصادی، اعتقادات مذهبی، ترجیح جنسی و رتبه شغلی مادران بر تمایل به داشتن فرزند دوم موثر بود (001/0>P ) و تنها متغیر ترجیح جنسی تأثیری بر تمایل به داشتن فرزند دوم نداشت (193/0=P). نتیجه‌گیری: بر اساس نتایج این تحقیق می‌توان گفت همچنان تمایل به داشتن فرزند دوم در بین زنان شاغل زیاد است. مهمترین عاملی که باعث تمایل و تقاضای برای داشتن فرزند دوم شده تنها نبودن، فرزند اول بوده است. بیشتر زنان در سنین زیر سی سال فرزند اول خود را به دنیا می‌آورند و تعداد فرزند مطلوب برای این زنان دو فرزند است.
